# A mathematical model for predicting the adult height of girls with idiopathic central precocious puberty: A European validation

**DOI:** 10.1371/journal.pone.0205318

**Published:** 2018-10-09

**Authors:** Pierre Lemaire, Gwénaëlle Duhil de Bénazé, Dick Mul, Sabine Heger, Wilma Oostdijk, Raja Brauner

**Affiliations:** 1 Univ. Grenoble Alpes, CNRS, Grenoble INP, G-SCOP, Grenoble, France; 2 Fondation Ophtalmologique Adolphe de Rothschild and Université Paris Descartes, Paris, France; 3 Diabeter, centre for pediatric and adolescent diabetes care and research, Rotterdam, The Netherlands; 4 Children’s Hospital Bult, Janusz-Korczak-Allee Hannover, Germany; 5 Department of Pediatrics, Leiden University Medical Center, Leiden, The Netherlands; Brown University Warren Alpert Medical School, UNITED STATES

## Abstract

**Background:**

A previous single-center study established a mathematical model for predicting the adult height (**AH**) in girls with idiopathic central precocious puberty (**CPP**).

**Objective:**

To perform internal and external validations by comparing the actual AH to the calculated AH established by this model and to update it.

**Methods:**

The original formula, calculated AH (cm) = 2.21 (height at initial evaluation, SD) + 2.32 (target height, SD) - 1.83 (luteinizing hormone/follicle-stimulating hormone peaks ratio) + 159.68, was established in a sample of 134 girls (group 4) and was applied to additional girls with CPP seen in the same center (group 1, n = 35), in Germany (group 2, n = 43) and in the Netherlands (group 3, n = 72). This formula has been updated based on these extended data, and both versions are available at the following location: **http://www.kamick.org/lemaire/med/girls-cpp15.html**.

**Results:**

Despite the differences among the 4 groups in terms of their characteristics at the initial evaluation and the percentages of patients treated with the gonadotropin-releasing hormone analogue, they have similar calculated and actual AHs. The actual AHs are 162.2±7.0, 163.0±7.6, 162.4±7.7 and 162.1±5.6 cm in groups 1 to 4, respectively. They are highly correlated with the AHs calculated by the formula established in the original group (group 4), with R at 0.84, 0.67 and 0.69 in groups 1 to 3, respectively. When the actual AHs and the AHs predicted by the Bayley and Pinneau method are compared, the R is 0.76, 0.51 and 0.64 in groups 1 to 3, respectively.

The absolute differences between actual AHs and the calculated AHs are greater than 1 SD (5.6 cm) in 15%, 35% and 28% of the patients in groups 1 to 3, respectively.

**Conclusion:**

This study validates and updates the previously established formula for predicting AH in girls with CPP. This updated formula can help clinicians to make treatment decisions.

## Introduction

Central precocious puberty (**CPP**) in girls is defined as the development of sexual characteristics before the age of 8 years due to the premature activation of the hypothalamic-pituitary-ovarian axis. In girls, CPP is idiopathic in more than 80% of cases [1–3]. Secondary to this activation, the secretion of estradiol increases the growth rate and accelerates bone maturation. These events can shorten the growing period, resulting in short adult height (**AH**). Treatment with a gonadotropin-releasing hormone (**GnRH**) analogue has been used to block the pituitary-ovarian axis and thus the estradiol secretion in girls with CPP for more than 30 years [4]. However, the question of whether to treat girls with idiopathic CPP remains. The reported height gain (AH-predicted AH at the onset of treatment, according to the Bayley and Pinneau method [5]) varies from 0.3 to 9.8 cm [6].

Given the lack of tools to determine who should be treated, we established mathematical models for predicting the AH and the age at first menstruation in a single-center study on 134 girls first seen between 1981 and 2007 for idiopathic CPP who reached their AH [7]. In this population, models were first established separately for the treated and untreated girls, but they were very similar and provided similar predictions for the entire group. Thus, we established a model for the entire population. The major limitation in this study was the lack of validation of the formulae on a separate population and external validation was necessary.

Consequently, the objective of the current study is to test this height prediction model on girls with idiopathic CPP who were seen during the next time period in the same center (internal validation) and in two other European groups (external validation), as well as to update the initial model. We therefore compared the actual AHs to the AHs calculated using the formula established by the model [7] in each of these three populations.

## Methods

### Ethics statement

All clinical investigations were conducted according to the principals expressed in the Declaration of Helsinki. In French patients, the Ethical Review Committee (Comité de Protection des Personnes, Ile de France III) approved the retrospective study [7] and stated that “This study appears to be in accordance with the scientific principles generally accepted and to the ethical standards of research. The study was lead in the respect of the French law and regulation” (Certificate AC 038). In the patients followed in the other centers, the data of the patients were anonymized and transferred to the coordinating center (See data availability transferred).

### Study design

We conducted a retrospective, multicenter cohort study. Five potential investigating centers were contacted if they had published CPP series and/or if they had previously collaborated with the coordinating center in Paris [3]. This study is reported according to the Transparent Reporting of a multivariable prediction model for Individual Prognosis or Diagnosis (TRIPOD) recommendations (S1 **Appendix**) [8].

### Participants

Two groups (Kiel, Germany and Rotterdam/Leiden, the Netherlands) agreed to participate to this validation. All locations are tertiary university pediatric hospitals centers. They respectively selected 43 and 72 girls with idiopathic CPP who reached their AH, defined as a growth during the preceding year of less than 1 cm in a menstruating girl. The coordinating center in Paris included 35 girls who were first seen for idiopathic CPP by the same senior pediatric endocrinologist (R. Brauner) between 2007 and 2011; none of the girls were included in the previous study [7]. The 35 girls did not differ from the original population of the coordinating center used to establish the formula, except for the percentage of treated girls, which was significantly greater in [7] than in the present study (78/134 vs 9/34, that is 58.2% vs. 25.7%, respectively, p<0.001).

The percentages of treated girls were 100% (43/43) and 97.2% (2/72 untreated) in the German (corresponding to the patients published in [9]) and Dutch (published in [10]) populations, respectively. Thus, all German patients seen during this period were treated with a GnRH analogue as part of a trial evaluating the safety and efficacy of this treatment in girls with CPP. As the models for treated and untreated girls were similar between them and with the entire population in terms of AH prediction in the previous study [7], the model was tested in each of the three populations and in the whole population.

### Outcome

We asked the two countries that agreed to participate in this validation to share the individual data with the following objectives: 1) to compare the populations between them, and 2) to include the parameters that compose the formula. The following data were thus extracted for each patient from existing databases or directly from patients’ files: parental heights (cm), age at onset of breast development, age at initial evaluation, height (cm), weight (kg), bone age (**BA**), peaks in luteinizing hormone (**LH**) and follicle-stimulating hormone (**FSH**) after the GnRH test, AH, whether MRI was obtained or not and treatment with GnRH analogues for more than 2 years or not. The height and body mass index (**BMI**) in SD scores (**SDS**), BA advance over chronological age in years and LH/FSH ratio were calculated automatically.

### Methods

CPP was diagnosed according to the appearance of breast development before the age of 8 years in all patients, accompanied by the presence of pubic or axillary hair, a growth rate greater than 2 SDS the year before their initial evaluation and/or a BA more than 2 years greater than their chronological age [11]. Organic intracranial lesions were excluded by MRI.

The BMI was calculated by the formula weight/height^2^. Height and BMI were expressed as SDS for chronological age with using French growth chart in all patients included [12,13]. The BA was assessed according to the Greulich and Pyle method [14]. The target height (**TH**) was calculated based on reported parental height [15]. The predicted AH was calculated with the Bayley and Pinneau method [5], with the exception of 9 cases whose predicted AH could not be calculated due to the absence of BA at the initial evaluation or because they had a BA of less than 7 years; we used the column “advanced” when the BA advance was greater than one year. The TH was unavailable in 2 German and 18 Dutch patients, among whom 10 had been adopted. The initial biological evaluation included basal and GnRH (100 μg/m^2^)-stimulated peaks of LH and FSH, except in 1 German and 9 Dutch patients. The serum samples were collected 0, 30, 60 and 90 min after the injection. In France, LH and FSH concentrations were measured using a two-site monoclonal immunoradiometric assay (LH-Coatria and FSH-Coatria; bioMerieux, SA, Marcy-l’Etoile, France). In Germany, LH and FSH concentrations were measured by radioimmunoassay or enzyme immunoassay at the Endocrine Laboratory of the University Department of Pediatrics of Kiel [9]. The GnRH analogue was depot triptorelin (D-Trp6-GnRH, Decapeptyl depot 3.75 mg i.m. every 4 weeks) in all cases.

### Statistical analysis methods

Data are expressed as the mean ± SD. Comparisons from groups to group are given as p-values of the Mann-Whitney U-test, and correlations are calculated according to Pearson’s definition. The calculated AH was compared to the actual AH on the basis of correlation, individual errors and absolute errors. We challenged three models of prediction of AH:

- A model established in a previous article [7] in the form of the following formula in SD and cm (available online at http://www.kamick.org/lemaire/med/girls-cpp15.html):
  ○ calculated AH (SD) = 0.39 (height at initial evaluation, SD) + 0.41 (TH, SD) - 0.33 (LH/FSH peak ratio)– 0.65.
  ○ calculated AH (cm) = 2.21 (height at initial evaluation, SD) + 2.32 (TH, SD) - 1.83 (LH/FSH peak ratio) + 159.68.
- The same model, updated on the entire dataset available:
  ○ calculated AH (cm) = 2.66 (height at initial evaluation, SD) + 1.71 (TH, SD) - 1.23 (LH/FSH peak ratio) + 158.08.
- The predicted AH, according to Bayley and Pinneau [5].

The data required to calculated AH are the age (years) and height (cm) at the initial evaluation, the father’s and mother’s heights (cm), and LH and FSH peaks.

## Results

### Comparison of the groups at the initial evaluation

In total, 150 patients of 3 countries participated to the study. We analyzed the data separately and then compared the characteristics among the 3 groups and to the original group of the coordinating center, which allowed for the establishment of the formula [7] (**Table 1**). The German patients were significantly younger at onset of puberty and at the initial evaluation, had greater BA advance and LH/FSH peak ratios, had lower predicted AH and were more frequently treated with the GnRH analogue than the French patients (recent and original group) and, to a lesser degree, than the Dutch patients, but the THs and the interval times between the onset of puberty and the initial evaluation were similar in all 3 groups.

**Table 1:** Comparison of the groups among each other and to the original group [7].

Characteristics	(1) French		(2) German		(3) Dutch		(4) Original		MWU-t (p-values)					
	n	mean ± SD	n	mean ± SD	n	mean ± SD	n	mean ± SD	1 vs 2	1 vs 3	1 vs 4	2 vs 3	2 vs 4	3 vs 4
Age at puberty onset. years	35	6.69 ± 0.72	43	5.47 ± 1.97	72	6.75 ± 1.38	134	6.63 ± 1.36	**<0.01**	0.10	0.17	**<0.01**	**<0.01**	0.59
Initial evaluation														
Age, years	35	7.60 ± 1.18	43	6.59 ± 1.85	72	7.83 ± 1.19	134	7.54 ± 1.41	**0.02**	0.12	0.47	**<0.01**	**<0.01**	0.19
Bone age advance, years	32	1.43 ± 1.31	42	3.05 ± 1.78	72	2.70 ± 0.90	131	1.50 ± 1.30	**<0.01**	**<0.01**	0.67	0.17	**<0.01**	**<0.01**
BMI, SDS	35	0.98 ± 1.53	43	1.87 ± 1.90	70	1.23 ± 1.62			**0.02**	0.44		**0.06**		
LH/FSH peaks ratio	35	1.32 ± 1.38	42	2.04 ± 1.37	63	2.72 ± 2.19	134	0.85 ± 0.86	**<0.01**	**<0.01**	0.12	0.09	**<0.01**	**<0.01**
Age at first menstruation, years	34	11.07 ± 1.49			70	12.40 ± 1.16	129	11.29 ± 1.24		**<0.01**	0.29			**<0.01**
Growth evolution														
Height. SDS	35	2.27 ± 1.28	43	2.86 ± 1.94	72	2.90 ± 1.45	134	2.11 ± 1.24	0.19	**0.04**	0.44	0.95	**0.01**	**<0.01**
Target height, cm	35	163.51 ± 5.39	41	163.98 ± 6.59	54	163.97 ± 7.10	132	161.74 ± 4.91	0.89	0.63	**0.05**	0.83	**0.05**	**0.01**
Actual adult height, cm	34	162.25 ± 7.01	43	163.00 ± 7.60	70	162.36 ± 7.69	134	162.11 ± 5.61	0.72	1.00	0.93	0.70	0.60	0.92
Calculated adult height, cm														
According to updated formula	35	162.55 ± 4.62	40	163.6 ± 6.32	49	163.14 ± 5.72	132	162.17 ± 4.19	0.50	0.59	0.92	1.00	0.29	0.20
errors	34	-0.27 ± 3.82	40	-0.01 ± 5.55	47	0.34 ± 5.32	132	-0.05 ± 3.85	0.94	0.50	0.73	0.70	0.96	0.49
According to formula [7]	35	162.36 ± 5.03	40	162.82 ± 6.12	49	161.78 ± 6.08	132	162.13 ± 4.24	0.92	0.83	0.93	0.76	0.85	0.94
errors	34	-0.04 ± 3.87	40	0.78 ± 5.62	47	1.67 ± 5.94	132	-0.01 ± 3.74	0.57	0.07	0.89	0.40	0.58	**0.01**
Predicted adult height [5]	31	163.6 ± 9.09	38	154.55 ± 9.76	54	160.91 ± 7.65	122	161.91 ± 7.98	**<0.01**	0.21	0.52	**<0.01**	**<0.01**	0.34
errors	30	-0.84 ± 5.99	38	8.10 ± 8.88	52	2.32 ± 6.65	122	-0.05 ± 6.81	**<0.01**	0.02	0.31	**<0.01**	**<0.01**	**0.03**

### Model performance

The formula established to calculate the AH on the original population [7] was applied to the 3 native populations. The actual AHs were compared to the calculated AH by this formula for each patient in each population and to the AHs predicted by the Bayley and Pinneau method.

**Fig 1:**
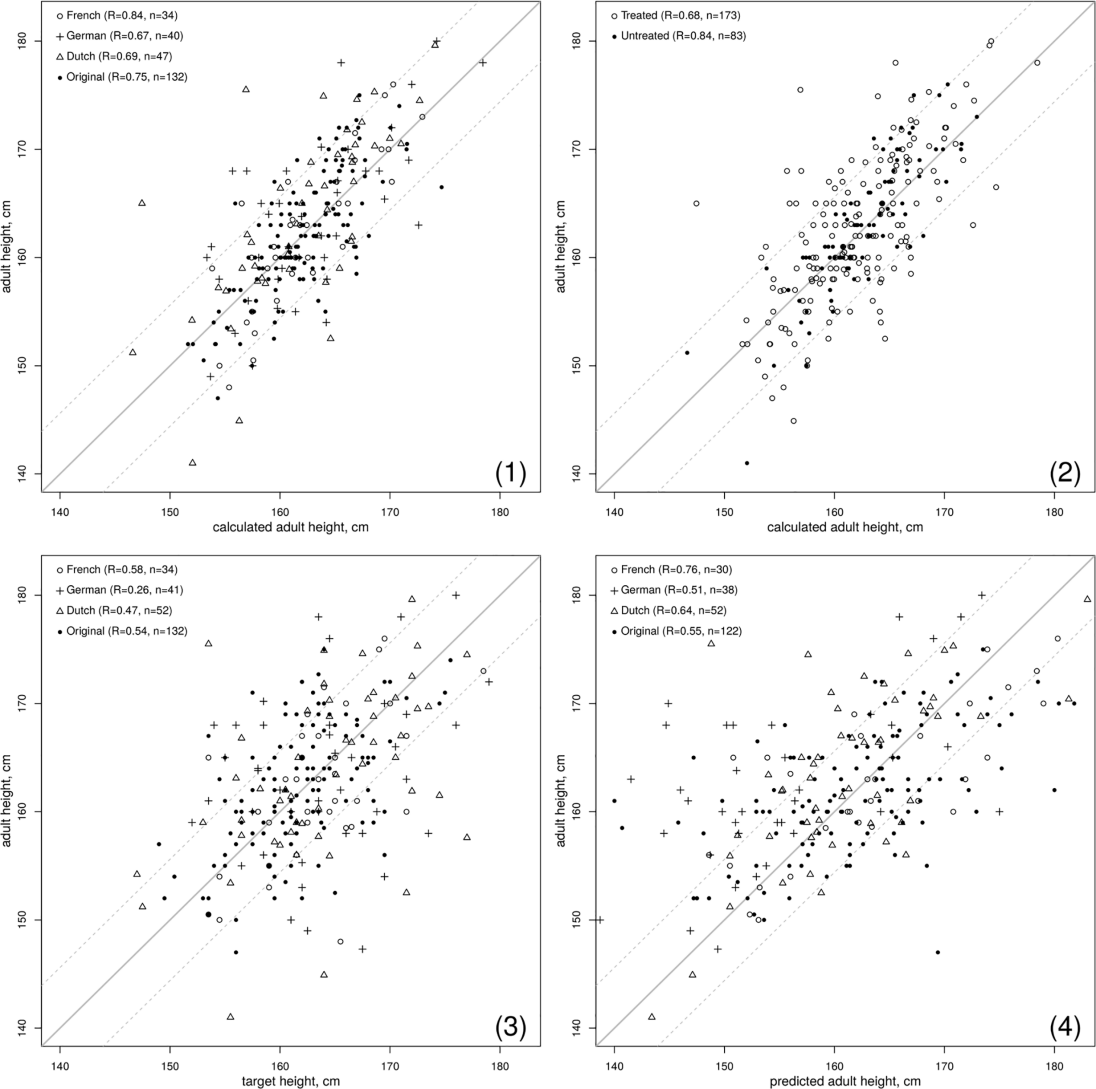
Correlation between actual adult height and calculated adult height in girls with idiopathic CPP. (1) and (2) calculated adult height are computed according to the formula established in the original group [7] and available at http://www.kamick.org/lemaire/med/girls-cpp15.html; as a basis for comparison actual adult height is also correlated to (3) target height and (4) adult height predicted by the Bayley Pinneau [5]. The plain line represents the reference for perfect prediction (calculated = actual), and the dotted lines represent ± 1 SD from that value.

The actual AHs and the AHs calculated at the initial evaluation by the formula, are not different in all groups including the original group (**Table 1**). The errors also are not different. The actual and the AHs calculated at the initial evaluation by the formula are highly correlated between them (**Table 2 and Fig 1**). In comparison, the predicted AHs obtained using the method of Bayley and Pinneau are significantly less correlated with the actual AHs and make significantly larger absolute and larger errors.

**Table 2:** Comparison of the actual and the calculated adult heights.

Indicator	(1) According to	(2) According to	(3) According to	MUW-t (p-values)		
	updated formula	formula from [7]	Bayley & Pinneau [5]	1 vs 2	1 vs 3	2 vs 3
all together						
R	0.75 [0.66:0.82]	0.71 [0.61:0.79]	0.57 [0.44:0.68]	0.88	0.04	0.24
Error. cm	0.05 ± 4.99 [-12.62:16.34]	0.90 ± 5.33 [-12.10:18.60]	3.36 ± 8.02 [-15.00:30.50]	<0.01	<0.01	<0.01
Absolute error, cm	3.79 ± 3.23 [0.08:16.34]	4.09 ± 3.51 [0.06:18.60]	6.46 ± 5.80 [0.00:30.50]	0.07	<0.01	<0.01
Over + Under estimations > 1SD	15 + 13 = 28 (23%)	12 + 20 = 32 (26%)	12 + 43 = 55 (46%)			
Over + Under estimations > 2SD	4 + 3 = 7 (6%)	2 + 4 = 6 (5%)	1 + 17 = 18 (15%)			
Over + Under estimations > 3SD	0 + 0 = 0 (0%)	0 + 2 = 2 (2%)	0 + 8 = 8 (7%)			
French						
R	0.86 [0.74:0.93]	0.84 [0.70:0.92]	0.76 [0.55:0.88]	0.94	0.68	0.81
Error. cm	-0.27 ± 3.82 [-8.20:6.59]	-0.04 ± 3.87 [-7.37:8.48]	-0.84 ± 5.99 [-10.80:14.20]	0.62	0.76	0.71
Absolute error, cm	3.02 ± 2.29 [0.09:8.20]	3.08 ± 2.29 [0.06:8.48]	4.68 ± 3.74 [0.00:14.20]	0.61	0.08	0.06
Over + Under estimations > 1SD	4 + 3 = 7 (21%)	2 + 3 = 5 (15%)	5 + 5 = 10 (33%)			
Over + Under estimations > 2SD	0 + 0 = 0 (0%)	0 + 0 = 0 (0%)	0 + 1 = 1 (3%)			
Over + Under estimations > 3SD	0 + 0 = 0 (0%)	0 + 0 = 0 (0%)	0 + 0 = 0 (0%)			
German						
R	0.68 [0.47:0.82]	0.67 [0.45:0.81]	0.51 [0.22:0.71]	0.96	0.63	0.69
Error. cm	-0.01 ± 5.55 [-11.34:12.29]	0.78 ± 5.62 [-10.21:12.45]	8.10 ± 8.88 [-15.00:30.50]	<0.01	<0.01	<0.01
Absolute error, cm	4.41 ± 3.30 [0.08:12.29]	4.53 ± 3.34 [0.21:12.45]	9.46 ± 7.36 [0.30:30.50]	0.92	<0.01	<0.01
Over + Under estimations > 1SD	6 + 5 = 11 (28%)	5 + 9 = 14 (35%)	1 + 23 = 24 (63%)			
Over + Under estimations > 2SD	2 + 2 = 4 (10%)	0 + 2 = 2 (5%)	1 + 13 = 14 (37%)			
Over + Under estimations > 3SD	0 + 0 = 0 (0%)	0 + 0 = 0 (0%)	0 + 6 = 6 (16%)			
Dutch						
R	0.76 [0.61:0.86]	0.69 [0.50:0.82]	0.64 [0.44:0.77]	0.89	0.66	0.95
Error, cm	0.34 ± 5.32 [-12.62:16.34]	1.67 ± 5.94 [-12.10:18.60]	2.32 ± 6.65 [-10.90:26.70]	<0.01	0.02	0.46
Absolute error, cm	3.83 ± 3.67 [0.09:16.34]	4.44 ± 4.24 [0.11:18.60]	5.29 ± 4.61 [0.30:26.70]	0.02	0.06	0.29
Over + Under estimations > 1SD	5 + 5 = 10 (21%)	5 + 8 = 13 (28%)	6 + 15 = 21 (40%)			
Over + Under estimations > 2SD	2 + 1 = 3 (6%)	2 + 2 = 4 (9%)	0 + 3 = 3 (6%)			
Over + Under estimations > 3SD	0 + 0 = 0 (0%)	0 + 2 = 2 (4%)	0 + 2 = 2 (4%)			

R is Pearson’s correlation with the actual adult height, given as estimated value with 95% confidence interval. Errors are given as mean ± SD [min:max].

Table 2 provides the detailed results on each group. On the entire data, the actual AH is overestimated by more than 1 SD (5.6 cm) by the mathematical model in 12 patients, and is underestimated by more than 1 SD in 20 patients; the corresponding values using the Bayley and Pinneau methods are 12 and 43 respectively. These patients with an average absolute error between the actual and the calculated AHs greater than 1 SD according to the original formula [7] were compared to the others; they are not different, except for the treatment rate (93.7% vs 71.9%, p = 0.02 for a Chi^2^-test).

The patients whose actual AH is overestimated by more than 1 SD (n = 12) were significantly different from the others as they had a shorter actual AH (152.8±6.31 vs 163.13±6.43 cm, p<0.001) as well as AH predicted by Bayley and Pinneau method (151.36±8.08 vs 160.51±9.17, p = 0.004), greater BA advance (3.24±1.65 vs 2.36±1.51 years, p = 0.04) and greater BMI (2.57±2.30 vs 1.31±1.64, p = 0.07).

The patients whose actual AH is underestimated by more than 1 SD (n = 20) differ from the others only by having greater actual AH (169.93±5.61 vs 163.13±6.43 cm, p<0.001).

The AH is overestimated (respectively underestimated) by more than 2 SD by the mathematical model in 2 (respectively 4) girls and by the Bayley and Pinneau model in 1 (respectively 17) girls.

### Model updating

A new formula was therefore established on the entire population of 284 girls, including the original (n = 134) and native (n = 150) populations. It was similar to the original formula and gave similar results in terms of AHs (R > 0.97 for all groups). The new, updated model is available and can be tried online at the updated **http://www.kamick.org/lemaire/med/girls-cpp15.html**.

## Discussion

Despite the differences in the initial characteristics established around one year after the onset of breast development and in the percentages of patients treated with the GnRH analogue between the original group (used to establish the formula) and the three native groups, the actual and calculated AHs are similar in the four groups. The model established in the original population gave a coefficient of correlation between the actual and the calculated AHs that was better in the internal validation but that was slightly lower and had more girls with absolute differences greater than 1 SD in the Dutch populations. However, its results are more accurate than those given by the Bayley and Pinneau method. The original model has been updated by including all populations. This model can be used to predict the AH in girls with idiopathic CPP. The prediction of the AH, together with the prediction of the age at first menstruation, may help clinicians to make treatment decisions for patients. We recently optimized our previous formula for predicting age at first menstruation in untreated girls based on the LH/FSH peak ratio [7] by adding the serum inhibin B concentration.

### Limitations

This study has several limitations. It is retrospective study. The characteristics of the populations of each center, including the percentages of the treated girls, are different. However, despite these differences, the actual AHs are similar and were significantly correlated with the AHs calculated by the model at the initial evaluation.

Thus, the German and Dutch girls were significantly younger at the onset of puberty and at the initial evaluation, had greater BA advance and LH/FSH peak ratios, had lower predicted AH and were more frequently treated than the girls in the French population. The THs and interval times between the onset of puberty and the initial evaluation were similar in all 3 groups. We have no explanation for these differences, as the recruitment was similar. Patients with precocious pubertal development are sent directly by their physician or by their pediatrician to the pediatric endocrinologist at the hospital. We have previously shown that the model for the prediction of AH gives similar predictions for treated and untreated girls and for the entire population [7]; thus, it was applied for all patients.

### Interpretation

The formulae established to predict the AH at the initial evaluation include the height of each parent, height at the initial evaluation, and the LH/FSH peaks ratio. This information is not surprising because the TH is a major determinant of AH in children with and without growth diseases. The height at the initial evaluation integrates the height level before and after the acceleration of the growth rate. We have previously shown that the LH/FSH peaks ratio after the GnRH test is significantly increased with the number of signs of puberty associated with breast development, and that the girls with a family history of early puberty had a significantly greater frequency of pubertal LH/FSH peaks ratios [16]. This finding suggests that the maturation of the hypothalamic-pituitary-ovarian axis depends on genetic factors, which impacts the evolution of the CPP. Guaraldi et al. [17] conducted an extensive analysis of the results on GnRH analogue treatment on the AH in CPP. Many papers reported that elevated height (or height SDS) before and at the withdrawal of treatment, as well as high TH, has also been positively associated with AH, supporting the primary influence of genetic factors in the determination of AH even in patients treated with the GnRH analogue.

The AH was significantly greater than that predicted by the Bayley and Pinneau method [5] in the German population and, to a lesser degree, in the Dutch population, but not in the Paris population used to establish the formula. This suggests that the GnRH analogue treatment, given to all but 2/115 girls of the external validation because of a low predicted AH, prevented the deterioration of the AH. This treatment was given to only 9/34 girls of the internal validation of the present study, the 25 others having a predicted AH higher than 155 cm. These differences are probably explained by the respective frequencies of rapidly and slowly progressing forms [11,18] different in the external and internal validation populations. In our previous study [7], we concluded that the similarity of the formulae for both treated and untreated groups suggests that the GnRH analogue treatment had no significant effect on the AH. However, the criteria used to select patients to be treated suggest that the treatment prevents the deterioration of AH in cases with rapidly evolving forms of CPP.

In a recent study, Lopes et al. [19] applied our mathematical model to 48 girls with idiopathic CPP. Their characteristics are similar to those of the 78 treated included in our model [7], except for their lower mean age at onset of puberty (5.75 vs 6.42 years) and greater interval time between the onset of puberty and treatment (2 vs 1.42 years). They similarly found that the treatment preserved the height potential. At diagnosis, the correlation between the actual and predicted AHs using the Bayley and Pinneau method was weaker than that using our model (r = 0.45 vs 0.58). They found better correlation (r = 0.74) when they compared actual AH to AH predicted by Bayley and Pinneau method after treatment, which is not surprising as the prediction improves when the age of the patient increases. This again suggests that the mathematical model should be used at the initial evaluation.

### Implications

Treatment of idiopathic CPP with GnRH analogues has been used for more than 30 years and is effective at regressing the pubertal signs. Several articles have shown that this treatment is well tolerated and has very few side effects [4,17,20,21]. A new dosage form of a once-yearly Histrelin subcutaneous implant has been developed, and this seems to be as effective as the injectable form [22]. The treatment effect on height gain is very controversial [6]. A review of the literature suggests that GnRH analogue treatment can influence height gain, but it is highly variable and essentially depends on age at the start of treatment, initial height, BA advance, and pubertal stage [6,7]. In the present study, the patients whose actual AH is lower than the calculated AH by more than 1 SD had greater BA advance and BMI than the others. We have previously shown [16] that among the girls with CPP, obese (BMI over 2 SDS) girls more often had a significantly higher BA advance of 2 years or more.

## Conclusions

The occurrence of CPP in a given girl exposes her to decreases in growth potential and age at first menstruation. The current study allows us to validate the previously established formula for predicting AH in girls with idiopathic CPP. We use this formula in our daily practice to predict at the initial evaluation the adult height to help us to take decisions on the treatment.

## Supporting information

S1 Appendix TRIPOD checklist: Prediction model validation. (PDF)

S1 File The complete data. This is a CSV file containing all the anonymized data use in this article and ready to be imported in any spreadsheet or data-analysis software.
(CSV)
